# Mesenchymal Stem/Stromal Cells Derived from Human and Animal Perinatal Tissues—Origins, Characteristics, Signaling Pathways, and Clinical Trials

**DOI:** 10.3390/cells10123278

**Published:** 2021-11-23

**Authors:** Magdalena Kulus, Rafał Sibiak, Katarzyna Stefańska, Maciej Zdun, Maria Wieczorkiewicz, Hanna Piotrowska-Kempisty, Jędrzej M. Jaśkowski, Dorota Bukowska, Kornel Ratajczak, Maciej Zabel, Paul Mozdziak, Bartosz Kempisty

**Affiliations:** 1 Department of Veterinary Surgery, Institute of Veterinary Medicine, Nicolaus Copernicus University in Torun, 87-100 Torun, Poland; magdalena.kulus@umk.pl (M.K.); kornel@umk.pl (K.R.); 2 Department of Histology and Embryology, Poznan University of Medical Sciences, 60-781 Poznan, Poland; 75094@student.ump.edu.pl (R.S.); k.stefanska94@o2.pl (K.S.); 3 Division of Reproduction, Department of Obstetrics, Gynecology, and Gynecologic Oncology, Poznan University of Medical Sciences, 60-535 Poznan, Poland; 4 Department of Basic and Preclinical Sciences, Institute of Veterinary Medicine, Nicolaus Copernicus University in Torun, 87-100 Torun, Poland; maciejzdun@umk.pl (M.Z.); maria.wieczorkiewicz@umk.pl (M.W.); hpiotrow@ump.edu.pl (H.P.-K.); 5 Department of Toxicology, Poznan University of Medical Sciences, 60-631 Poznan, Poland; 6 Department of Diagnostics and Clinical Sciences, Institute of Veterinary Medicine, Nicolaus Copernicus University in Torun, 87-100 Torun, Poland; jmjaskowski@umk.pl (J.M.J.); dbukowska@umk.pl (D.B.); 7 Division of Anatomy and Histology, University of Zielona Gora, 65-046 Zielona Gora, Poland; m.zabel@wlnz.uz.zgora.pl; 8 Prestage Department of Poultry Science, North Carolina State University, Raleigh, NC 27695, USA; pemozdzi@ncsu.edu; 9 Department of Anatomy, Poznan University of Medical Sciences, 60-781 Poznan, Poland

**Keywords:** perinatal mesenchymal stem/stromal cells, MSCs differentiation, signaling pathways

## Abstract

Mesenchymal stem/stromal cells (MSCs) are currently one of the most extensively researched fields due to their promising opportunity for use in regenerative medicine. There are many sources of MSCs, of which cells of perinatal origin appear to be an invaluable pool. Compared to embryonic stem cells, they are devoid of ethical conflicts because they are derived from tissues surrounding the fetus and can be safely recovered from medical waste after delivery. Additionally, perinatal MSCs exhibit better self-renewal and differentiation properties than those derived from adult tissues. It is important to consider the anatomy of perinatal tissues and the general description of MSCs, including their isolation, differentiation, and characterization of different types of perinatal MSCs from both animals and humans (placenta, umbilical cord, amniotic fluid). Ultimately, signaling pathways are essential to consider regarding the clinical applications of MSCs. It is important to consider the origin of these cells, referring to the anatomical structure of the organs of origin, when describing the general and specific characteristics of the different types of MSCs as well as the pathways involved in differentiation.

## 1. Human and Animal Perinatal Tissues—Anatomical, Histological, and Cellular Characteristics

Perinatal tissues are an extremely rich source of various cell types. The placenta is an organ that is irreplaceable for the development of the fetus, because it is a multicellular barrier. The placenta is also unique in terms of the origin of the cells that form it, because it is composed of cells of maternal and fetal origin, that are genetically distinct organisms [1]. The necessary exchange of nutrients, metabolites, and endocrine regulation must take place simultaneously with the maintenance of immunological tolerance between the two organisms [2]. It replaces some of the inactive organs in the fetus (lungs, liver, endocrine glands). The placenta is formed after successful fertilization and implantation of the embryo in the mammalian uterus. There is a distinction between the maternal-uterine part, which is the endometrium, and the fetal part. The membrane of maternal origin is referred to as decidua. Decidualization occurs at different rates depending on the type of placenta, and is stimulated by factors secreted by the blastocyst (histamine, prostaglandins). The *decidua basalis* is the part of the endometrium immediately adjacent to the fetal bladder and is involved in the formation of the chorioallantoic placenta. The *decidua capsularis* separates the fetal bladder from the uterine cavity, and the *decidua parietalis* connects the *decidua basalis* and *decidua capsularis*.

Fetal membranes arise from the zygote, and function as accessory organs. The fetal membranes consist of the yolk sac (*saccus vitellinus*), amnion, chorion, and allantois. As the early stage embryo migrates through the fallopian tube where fertilization occurred, the cells divide to form a morula (*morus*) and then a blastocyst. The embryonic node (*massa embryonica*) is where the embryo proper forms, and the peripherally arranged flattened cells make up the trophoblast [3]. The trophoblast cells have microvilli where the cells of the tubular epithelium of the endometrium fuse. The trophoblast cells contain proteolytic enzymes (zinc-containing metalloproteases) that degrade the endometrial epithelium, allowing the blastocyst to penetrate deep into the endometrium [4]. Initially, the trophoblast has two layers: an inner layer (*cytotrophoblastus*) and an outer layer (*syncytiotrophoblastus*). The inner layer consists of highly proliferating mononuclear cells, and the outer layer is formed by cell fusion and has invasive capacity and is responsible for anchoring the blastocyst in the uterus [5]. It will then develop into the chorion and participate in the construction of the placenta.

The yolk sac forms as the first fetal membrane. The wall of the yolk sac is trilaminar (*saccus vitellinus trilaminaris*) due to the ingrowth of the extraembryonic mesoderm between the trophoblast and the extraembryonic endoderm. The yolk sac has hematopoietic functions because the first blood cells and blood vessels are formed in it [6]. Primary germ cells (gonocytes) also appear in the wall of the yolk sac, which then migrate to the gonadal primordia [7]. In the further development of the embryo, the yolk sac usually disappears and the established pedicle, together with the yolk vessels and the surrounding mesoderm, become part of the umbilical cord.

The membrane that directly covers the embryo is the amnion, which is formed around day 7 of embryonic development. The space between the amnion and the embryo, the amniotic cavity, is filled with amniotic fluid (*liquor amnioticus*) [8]. The amnion consists of ectodermal epithelium and mesenchymatous tissue and is generally not vascularized. The amniotic epithelium is composed of large, polygonal flat cells, the surfaces of which are covered with microvilli [9]. These cells may exfoliate into the amniotic fluid. Prenatal diagnosis utilizes exfoliated epithelium by collecting fluid through a puncture of the amniotic cavity (amniocentesis) [10]. Between individual amniotic epithelial cells, on their lateral surfaces, intercellular spaces are formed. They are filled with microvilli and protuberances, named amniotic water vacuoles because of their appearance. These cells contain numerous lipid droplets and glycogen grains. The basement membrane of the amniotic epithelium contains numerous reticular fibers, passing into mesenchymatic tissue, which is rich in fibroblastic cells and collagen fibers [11]. This tissue shows great strength. Amniotic fluid is produced by epithelial cells, and in addition serous fluid is infiltrated from the mesenchyme through the intercellular spaces. The amount of fluid changes during pregnancy, and the main components are water (99%), saccharides, proteins, urea, and also exfoliated cells or fetal downy hairs (*lanugo*). Amniotic fluid is constantly and rapidly exchanged. Resorption occurs by the amniotic epithelial cells and by the fetus [12]. Amniotic fluid has important functions in providing the embryo with a watery environment, protection from injury, amortization, and metabolism.

The chorion lies in direct contact between the amnion and the endometrium, forming an integral part of the placenta. It arises from the trophoblast, which merges with the extraembryonic mesoderm [13]. The surface of the chorion is covered by characteristic villi, which come into close physical contact with the uterine endometrium [14]. The shape of these villi varies depending on the type of implantation and placenta, and the animal species. The chorion does not produce blood vessels and vascularization comes from the allantois or yolk sac. The chorion over a large area fuses with the allantois to form the *chorioallantois* [15].

The last fetal membrane to form is the allantois, which is of endo- and mesodermal origin. It arises from the posterior part of the primary intestine. Already at an early stage, hematopoietic islands and blood vessels form in the wall of the allantois, which make up the initial formation of the umbilical artery and vein [16]. The intraembryonic part of the allantois merges with the bladder valve and then disappears to form the urachus and then the median umbilical ligament [17]. The main role of the allantois is to supply blood vessels to the chorion, forming the placental circulation. In some animals, the allantois also has a role related to excretion of metabolic products, in which case it is well developed and large in size [18]. In humans and rodents, it has a residual form as the diverticulum and *caulis allantoicus*.

The umbilical cord (*funiculus umbilicalis*) extends from the ventral wall of the embryo and connects the embryo to the placenta. It includes the yolk and umbilical blood vessels, the yolk duct, and the *caulis allantoicus*, surrounded by the dermal cord. The yolk duct quickly overgrows and becomes a solid string of cells with yolk vessels. The allantois duct is lined with a flat monolayer of epithelium [19]. Anastomoses may be formed between the vessels and the course of the vessels forms a spiral, providing great flexibility [20]. The mesoderm of all the ducts running in the umbilical cord fuses together and develops into a mucous connective tissue (*tela mucoidea connectens*), otherwise known as Wharton’s jelly. It contains abundant intercellular substance, rich in glycosaminoglycans, collagen, elastic and reticular fibers, and fibroblasts. Externally, the umbilical cord is surrounded by a thin monolayer of epithelium (periderm) of ectodermal origin [21]. The umbilical cord may contain nerves that receive sensory stimuli related to tissue tension [22]. The cord varies in strength depending on the species, but is easily broken during birth. Bleeding from the cord vessels is not abundant due to contraction of the strong arterial muscle layer and rupture of the venous connection to the placenta.

Among mammals, a wide range of placental strategies can be observed based upon the different gestational and environmental needs of the fetus [23]. Mammalian placentas are mostly classified into two types: yolk sac placenta and chorioallantoic placenta. The yolk sac placenta is a trilaminar yolk sac attached to the uterine tissue, which usually plays a role during the early post-implantation period. In most mammals, with the exception of rodents and rabbits, the yolk sac placenta becomes reduced after the first trimester of pregnancy [24]. Thus, impaired structural and functional development of the yolk sac contributes to embryo/fetal toxicity and teratogenicity in rats [25]. The chorioallantoic placenta is formed from the endometrium of the mother and the trophectoderm of the embryo, and is the principal placenta in mammals during middle to late gestation [24].

There are two main classifications of chorioallantoic placentas. The first, based on the distribution of villi over the surface of the chorion, divides placentas into diffuse, multi-cotyledonary, zonary, and discoid. In a diffuse type, such as those of pigs [26], camel [27], lemurs, and lorises [28], the surface is covered with villi that interdigitate with crypts in the uterine epithelium. The villi may aggregate into bundles, forming microcotyledons, such as those observed in horses. In turn, most ruminants produce cotyledonary placentas. Each cotyledon is a small disk, with their numbers varying from a few in deer (oligocotyledonary) to many in bovids (polycotyledonary) [29]. Furthermore, the zonary placenta is typical for carnivores, forming a belt around the chorionic sac [24]. Finally, the discoid placenta is characterized by a roughly circular area. This type of placenta is found in most primates, including humans, as well as in rodents and rabbits [24].

The second classification is based on the number of tissues separating maternal and fetal blood. In the hemochorial type of placenta, the trophoblast invades the uterine epithelium, stroma, and maternal arterial walls to come into direct contact with maternal blood [23]. There are hemomonochorial (higher primates, e.g., human; hystricomorph rodents, e.g., guinea pig), hemodichorial (rabbits), and hemotrichorial (most myomorph rodents, such as rats and mice) placentas, with one, two, and three trophoblast layers in the interhaemal barrier, respectively [24,28]. Furthermore, there is a clear difference between higher primates and lemurs or lorises (lower primates), with the former (similarly to pigs) producing the epitheliochorial type of placenta. This is the most superficial type of placenta, lacking significant invasion of the uterine lining [26,28]. Apart from these two types, i.e., hemochorial and epitheliochorial, there is also the endotheliochorial type. In this case, the maternal uterine epithelium and connective tissue disappear after implantation, and the trophoblasts come into direct contact with the maternal endometrium. This type occurs in all four major clades of eutherian mammals (Euarchontoglires, Laurasiatheria, Xenarthra, and Afrotheria), including carnivores [24].

## 2. Mesenchymal Stem/Stromal Cells—Origin, Cellular and Molecular Characteristics, and Signaling Pathways Involved in Differentiation

Cells with stem-like potential have been of continued interest to researchers in various fields for many years. Division and classification are still being attempted, and molecular characterization appears to be the most appropriate. Enabling mesenchymal stem/stromal cells (MSCs) cells to be used in regenerative medicine, especially for musculoskeletal diseases, degenerative diseases, or incurable conditions, is very promising. MSCs also promote immunomodulation as they can both inhibit and stimulate the immune system and express many immunosuppressors [30,31] and influence autophagy processes [32]. They also exhibit anti-apoptotic [33] and antioxidant [34] effects, which promote the treatment of neuromuscular soreness [35]. In recent years, there has been an abundance of progress in research related to the isolation and culture of multipotent stem cells derived from various human and animal tissues. The most commonly used sources of MSCs are: bone marrow, adipose tissue, cord blood, peripheral blood, muscle tissue, placenta, and amniotic fluid. According to the most general definition of MSCs, they need to exhibit the ability to adhere to plastic surfaces; express specific differentiation clusters, such as CD73, CD90, and CD105; and have the ability to differentiate into osteogenic, chondrogenic, or adipogenic lineage cells in vitro. Unlike cells of the hematopoietic lineage, they do not express CD14, CD34, CD45, and HLA-DR [36]. Additionally, MSCs can express other markers such as nestin (Tuj-1) for neural cells [37], smooth muscle α-actin, smooth muscle myosin heavy chain for muscle cells [38], or transforming growth factor-β (TGF-beta) receptor [39] and integrins [40]. It should be emphasized that cells expressing neural specific markers in in vitro cultures often present only the transient neuron-like morphology. Inducing full neuronal functionality remains an elusive goal. Cultured cells fail to generate functional polarity and form new signals passing neuronal synapses [41]. Undoubtedly, significant work remains to understand the biology of MSCs.

Initially, the discovery and identification of pluripotent embryonic stem cells (ESCs) [42] revealed the existence of cells that can self-renew indefinitely and differentiate into all three embryonic germ layers [43,44], revealing a wide field of applications. Ethical issues have limited the availability of new ES cells lines [45]. An alternative source of stem cells has proven to be adult tissues, which contain a certain pool of multipotent cells. The use of adult stem cells is devoid of ethical considerations, they are widely available, and there is a lower risk of tumorigenesis [46,47]. Hematopoietic stem cells (HSCs), which have the ability to differentiate into all lineages of the blood and immune system, have been distinguished. HSCs have found applications in the treatment of blood disorders and leukemia [48].

A promising source consists of MSCs isolated from adult tissues, which in vitro differentiated into adipogenic [49], chondrogenic [50], osteogenic [51], or even neurogenic lineages [52]. The cells taken from the patient are multiplied, differentiated, and have therapeutic applications. This would involve no need for xenotransplantation, as the cells would come from the same patient. However, tissue harvesting itself is associated with rather painful and invasive procedures and the possibility of infection at the harvest point [53]. In addition, the patients themselves who require cell therapy are not in well enough health to perform the procedure. The clinical condition of donor patients is also not without significance. It appears that the exhaustion of physiologically occurring MSCs in patients with primary osteoarthritis occurs. In biomechanical joint damage, subchondral bone populations of MSCs are not impaired [54,55,56]. It is also important to establish the ability to proliferate and differentiate, which are key processes in the harvesting of MSCs. It appears that cells obtained from adult tissues exhibit different potentials [57], and the capacity for proliferation and differentiation decreases during in vitro culture after successive passages [58]. Differentiation of cells toward the tissues from which they originate is overall more successful, as evidenced by the superior mechanisms of tissue-specific MSCs [59]. It was also found that the differentiation capacity, as well as the self-renewal potential, is dependent on the physiological state of the donor (age, health status), its genetics, and the influence of environmental conditions [60,61,62,63,64]. For example, the MSCs obtained from young rats presented faster growth correlating with the levels of proliferating cell nuclear antigen and higher glucose utilization compared to older ones [65]. The impact of telomere erosion in MSCs was not insignificant here either [66]. MSCs can be derived from perinatal tissues, which include the placenta [67], umbilical cord [68,69,70], or the cord blood itself [71]. In addition, fetal tissue [72] and the surrounding amniotic fluid [73] are promising sources for stem cell derivation. In principle, stem cells derived from perinatal and fetal tissues should have a greater potential for self-renewal and the ability to proliferate and differentiate; however, many sources have reported that they show considerable diversity [53,74], the details of which are given in the following sections describing the characteristics of individual perinatal MSCs.

Many authors have suggested that an attempt should be made to rename the MSC because the term “mesenchymal stem cells” has been overused by groups commercializing administration of “MSCs” for therapeutic purposes. However, the commercial entities do not perform differentiation of the “MSCs”, and the therapeutic effect of the administered compound is based mainly on local action and secretion of active factors [75]. Indeed, traumatically altered tissues call induce repair mechanisms. MSCs respond to these signals, migrate [76], and act by secreting active factors (immunomodulatory, trophic, regenerative) [77,78]. This translates into a local therapeutic effect that is, in fact, based on the specific stem cells of a given patient that have been activated by the exogenous MSCs administered [79,80]. Therefore, the first proposal for a new name for these cells in general is “medicinal signaling cells”, which reduces the misleading of patients that they are receiving typical stem cells that will produce new tissue [81]. There has also been a proposal (from the International Society for Cellular Therapy (ISCT)) that fibroblast-like cells, which are plastic-adherent, should be called multipotent mesenchymal stromal cells regardless of origin [82]. Attempts to undertake a uniform classification and nomenclature change related to MSCs have been ongoing for several years. The acronym “MSCs” is proposed to remain in the nomenclature, but the authors of individual studies should carefully specify the origin and demonstrate the functional properties of MSCs [83]. However, it is important to consider that stem/stromal cells remain the most widely used terms to describe MSCs. Nevertheless, the wide variation in differentiation potential, the lack of standardized acquisition procedures, and the absence of a single universal marker significantly limit standard clinical application. A major step is the collection itself, the isolation of MSCs from a donor, which influences the final cell population [84]. Most often, these are simple procedures involving mechanical fragmentation of the harvested tissue or enzymatic digestion, followed by seeding and attachment of the cell suspension into culture dishes. The explant-derived method involves breaking the tissue fragments into small pieces to facilitate diffusion of nutrients and gases, and placing these fragments in the culture medium. MSCs proliferate and colonize on the surface of the bottom of the dish. Enzyme-mediated isolation involves incubation and enzymatic digestion of the harvested tissue to release individual cells from the extracellular matrix (ECM), followed by centrifugation and placement of the resulting cell pellet in the culture medium. Enzymatic isolations are considered to be more efficient, but the explant method provides higher homology with better proliferation and viability rates [85,86]. It is suggested that is the superior properties of the explant-derived cells are associated with less stress on the cells [87].

The therapeutic effects of MSCs are related to the secretion of paracrine factors, including cytokines, growth factors, mRNAs, miRNAs, and signaling lipids. Much recent research has focused on nanoparticles derived from the cell membrane of MSCs, called exosomes [88,89]. These vesicles are secreted outside the cell, mediating cell-to-cell communication. MSC-derived exosomes are a tantalizing possibility for innovative cell-free therapies, which could have advantages over whole-cell therapies. Extracellular vesicles (EVs) can occur as exosomes released extracellularly by fusing with the cell membrane. EVs are also microvesicles that bud from the plasma membrane, and sometimes occur as apoptotic bodies of varying sizes [90]. In relation to secretory capacity, therapeutic properties are associated with immunomodulatory and trophic effects at the site of injury/disease [91], and EVs are able to penetrate the blood–brain barrier [92].

With recent advances in the study of exosome-based therapies with MSCs, it is important to assess their localization, tracking, and monitoring after transplantation in vivo [93]. One method is magnetic resonance imaging (MRI), which allows the localization of exosomes labeled with contrast agents (such as ultra-small superparamagnetic iron oxide nanoparticles) [94].

### 2.1. Different Origins of MSCs—Sources, Cell Characteristics, Possible Applications

MSCs can be isolated from any vascularized tissue, and studies have indicated that pericytes (perivascular cells) may be the source, as the gene expression profile of adipose tissue pericytes is remarkably similar to that of adipose tissue stem cells [95,96]. Bone marrow is rich in stem cells that show potential to differentiate into osteoblasts. Initiating the differentiation process through the release of transforming growth factor β1 (TGF-β1) leads to the development of osteocytes [97]. Research on the clinical application of various procedures using MSCs in bone regeneration has been ongoing and advanced for several years [98,99,100]. Supplementation of growth factors to promote bone repair by MSCs is also currently being investigated, particularly in relation to tissue-engineering scaffolds [101]. MSCs also provide opportunities for the treatment of intervertebral disc degeneration. Although they exhibit high regenerative potential, chondrogenic differentiation, and anti-inflammatory effects, the specific nature of the intervertebral disc structure (including lack of blood vessels, low pH and glucose levels, and hypoxia) largely limits the application of this therapy [102].

Stem cells derived from adipose tissue have great potential for treating orthopedic conditions [103,104]. Adipose-derived stem cells (ASCs) have been shown to have multidirectional differentiation properties, including into adipocytes, osteocytes, and chondrocytes, and they have been shown to express MSCs markers (CD 29, CD44, CD90) with a negative expression of hematopoietic markers (CD31, CD34, CD45). The demonstration of cartilage repair in an animal model was also significant [105]. MSCs can also be isolated from oral tissues such as dental pulp, gingiva, dental follicles, alveolar ligaments, and others. These cells are used in tissue engineering as they show potential for multidirectional differentiation and are easy to obtain and show regenerative potential [106]. MSCs derived from dental tissues, in addition to their ability to differentiate into the mesodermal lineage, have been shown to undergo ectodermal-neurocyte and endodermal-hepatocyte differentiation [107]. MSCs also show remarkable potential in regenerative processes and wound healing. MSCs extracted from the basal layer of the epidermis and hair follicles have been shown to promote skin healing, new blood vessel formation, and endothelial transformation. The mechanisms of these processes are not entirely clear [108]. MSCs in the treatment of cutaneous wounds inhibit inflammation, promote angiogenesis, and accelerate wound closure, and their action is based on paracrine mechanisms [109]. MSCs have also been shown to affect extracellular matrix remodeling [110,111]. Standard sources of MSCs (adipose tissue or bone marrow), as well as perinatal tissues, are effective sources to treat wounds [112,113].

Recently, there has also been research into the possibility of using MSCs from different sources to treat female infertility of various backgrounds [114]. Additionally, numerous literature data have indicated a high stemness potential of human [115], as well as animal [116], ovarian granulosa cells (GCs). GCs, co-forming the ovarian follicle, are classified as mulipotent cells [117,118] and play a key role in oocyte maturation through their regular contact. Their ability to differentiate into osteoblasts, chondroblasts [119], mioblasts [120], or even cells from the neurogenic lineage [121] has been experimentally demonstrated. However, a recent study showed that although there was a comparatively high expression of stemness markers of cells isolated from ovarian follicles, the osteogenic and adipogenic differentiation capacity in elderly patients was inferior to young ones [122].

Due to the many limitations in the use of adult MSCs, perinatal MSCs could be applicable. They show higher plasticity and proliferation capacity than adult MSCs [123]. They also differ from embryonic stem cells (ESCs) in that they express pluripotent markers and have active telomerase, but at a much lower level than ESCs [123]. As a result, their use is not associated with the risk of tumorigenesis, as in the case of ESCs [124,125]. Immunological aspects of the transplanted cells are also important [126]. Perinatal MSCs should be immunologically neutral due to the absence of intracellular HLA class II and poor expression of HLA class I compared to adult MSCs [127]. Perinatal cells are also distinguished from adult MSCs by their greater capacity for osteogenesis in vitro, as well as in vivo [128], exhibiting greater osteogenic potency [129]. Once perinatal MSCs are obtained, they are centrifuged and cultured in a medium containing serum. Upon adhesion to the plastic substrate, they form colonies and the cells assume a spindle shape, resembling fibroblasts. Perinatal MSCs should not express hematopoietic and endothelial markers [130]. However, these are only general definitions of MSCs, which have been verified by subsequent findings in the stemness field. Simply showing expression of molecular markers should not determine that the cells in concern are pluripotent. Proteins, meaning products rather than the genes themselves, are actually responsible for the mechanisms and abilities of pluripotency [131]. For example, it is the amount of Oct-4 protein that determines the maintenance of pluripotency and also the direction of cell differentiation [132,133]. Therefore, phenotypic characteristics, as well as the degree and efficiency of differentiation of the cells studied, should be used to demonstrate their pluripotent abilities.

### 2.2. Differentiation of MSCs—Regulatory Factors and Signaling Pathways

**Figure 1:**
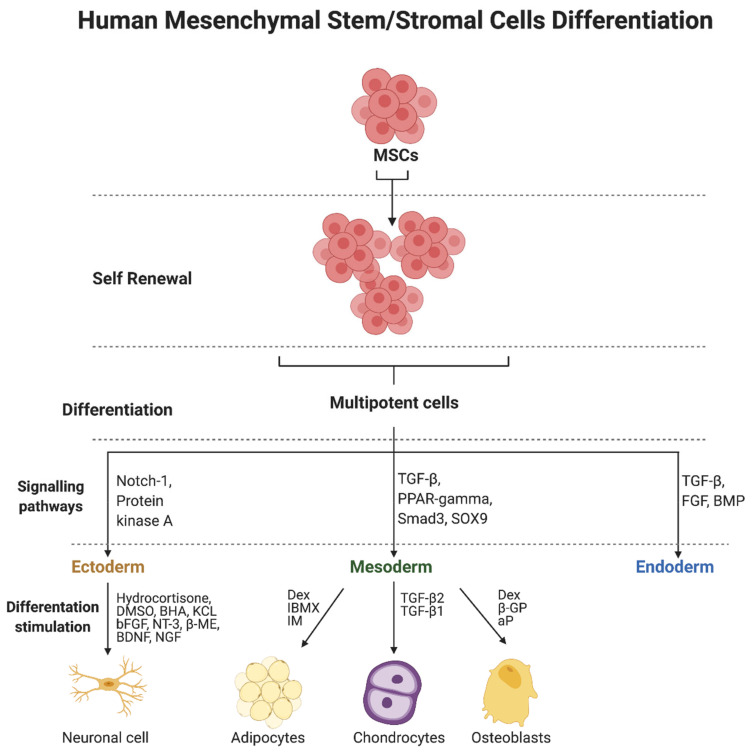
Human mesenchymal stem/stromal cells (MSCs) differentiation and signaling pathways. Abbreviations: aP—ascorbic acid phosphate; β-GP—β-glycerophosphate; β-ME—β-mercaptoethanol; BDNF—brain-derived neurotrophic factor; bFGF—basic fibroblast growth factor; BHA—butylated hydroxyanisole; BMP—bone morphogenetic protein; Dex—dexa-methasone; DMSO—dimethyl sulfoxide; FGF—fibroblast growth factor; IBMX—isobutyl-methylxanthine; IM—indomethacin; KCL—potassium chloride; NGF—nerve growth factor; Notch1—notch homolog 1; NT-3—neurotrophin-3; PPAR—gamma-peroxisome proliferator-activated receptor gamma; Smad3—mothers against decapentaplegic homolog 3; SOX9—transcription factor SOX9; TGF-β—transforming growth factor-β. Created with BioRender.com (accessed on 5 October 2021).

Genetic regulation and transcription factors are involved in the differentiation of MSCs. In addition, the microenvironment itself can promote proliferation and differentiation, in addition to providing conditions for growth [134,135,136]. Differentiation towards osteogenesis, adipogenesis, or chondrogenesis is, in theory, straightforward for MSCs as they are derived from the same embryonic lineage. However, theory alone does not correlate to the actual laboratory results. The differentiation abilities are highly dependent on the source of the starting cells, the primary cell population, and also the direction of differentiation [137]. A mixture of dexa-methasone (Dex) isobutyl-methylxanthine (IBMX) and indomethacin (IM) is used to induce adipogenesis (Figure 1). Confirmation of a properly occurring differentiation process has been provided by Oil Red O staining of neutral triglycerides and lipids in cells and visualization of lipid droplets [138]. Osteogenic differentiation is performed by stimulation with dexa-methasone (Dex), β-glycerophosphate (β-GP), and ascorbic acid phosphate (aP) [139,140]. Alkaline activity and calcium accumulation were analyzed to confirm the process. TGF-β2 and TGF-β1 are used for chondrogenesis [141]. Pathways involving TGF-β, PPAR-gamma, Smad3, and SOX9 are involved in differentiation into the mesodermal lineage [142,143,144], whereas ectodermal differentiation into the neurogenic lineage involves the Notch-1 pathway and the protein kinase A (PKA) pathway [145,146,147]. Hydrocortisone, DMSO, BHA, and KCL, as well as bFGF, NT-3, β-mercaptoethanol (β-ME), BDNF, and NGF are used to induce differentiation [148,149]. Evaluation of differentiation was based on Tuj-1, γ-aminobutyric acid (GABA), MAP-2, neurofilament 200, among others [150]. Signaling pathways involving TGF-β, fibroblast growth factor (FGF), and bone morphogenetic protein (BMP) are involved in endodermal differentiation of MSCs [151].

Research continues to refine the procedures involved in the processes of stem cell differentiation. Transcription factors for differentiation, such as osterix/Sp7 (Osx), runt-related transcription factor 2 (RUNX2), and Dlx5 are known to play an important role in osteogenic differentiation [152,153]. They influence the course of various signaling pathways that play a role in osteogenic differentiation processes, including WNT [154], BMP [155], and Akt [156]. They may act as regulators as they both activate and inhibit the differentiation of MSCs. Additionally, a recently published study demonstrated the accelerating effect of using nanocomposites that activate the WNT/β-catenin pathway in osteogenic differentiation of MSCs [157].

It is noteworthy that the signaling pathways and factors involved in cell differentiation in a specific cell line described above are not obligatory and may proceed in different ways. For example, the study by Brady et al. [158] compared factors that promote chondrogenesis using human MSCs derived from perinatal and adult bone marrow. TGFβ3 was shown to induce SMAD3 phosphorylation in adult BM-MSCs but not in fetal BM-MSCs. It was also observed that the induction of chondrogenesis in adult BM-MSCs occurred under the influence of TGFβ3 but not by BMP2. Conversely, differentiation of fetal BM-MSCs was induced by BMP2 but not TGFβ3. Further, fetal BM-MSCs stimulated chondrogenesis simultaneously when TGFβ3 and BMP2 were used. It was shown that they produced tissue with proteoglycan and type II collagen content similar to that produced by adult BM-MSCs treated with TGFβ3 alone [158].

## 3. Mesenchymal Stem/Stromal Cells Derived from Human and Animal Perinatal Tissues

MSCs are present in tissues of fetal origin in both animals and humans. Over time, their isolation from tissues of fetal origin has become feasible using well-described laboratory protocols. Isolation of stem cells from fetal material does not raise ethical dilemmas due to the fact that these tissues are treated as medical waste immediately after delivery. As a result, the material for laboratory analyses is readily available [159]. Research material can be taken from tissues obtained from invasive diagnostic and treatment procedures throughout the pregnancy, planned terminations, and after the full-term vaginal delivery or cesarean section. The studies focused on perinatal stem cells are conducted worldwide. Several countries, such as the U.S. or China, released their specific cellular and gene therapy guidance and established regenerative medicine products’ regulations [160,161]. Each European Union member state currently has its own specific regulations targeted on embryonic stem cells and perinatal tissues research. Perinatal stem cell clinical trials must be approved by the local bioethical commissions and be conducted in line with the EU clinical trials registration law [162,163]. Cells of fetal origin, also known as perinatal stem cells, are derived from extraembryonic structures, such as the placenta, umbilical cord, and amniotic fluid [164,165]. Perinatal tissues are also widely used as a reservoir of hematopoietic progenitor cells obtained from the umbilical cord blood [166]. Furthermore, it is believed that the perinatal stem cells obtained at the very early stages of pregnancy (first trimester) possess a higher regenerative potency compared to cells isolated from full-term or near-term feto-maternal tissues. Several authors reported that they detected the higher expression of pluripotency genes in the samples collected at the earlier stages of pregnancy [167,168,169]. However, the others found no differences in their expression throughout the gestation [170].

Over the recent years, numerous studies have focused on perinatal stem cells applications in clinical practice and analyzed their characteristics. In most cases, to reach the appropriate number of cells before the MSCs transplantation, they should be cultured in vitro for several passages. It raises the concern about the occurrence of replicative senescence, which could affect the clinical effectiveness of cellular therapies. Importantly, it has been noted that the perinatal MSCs seem to be less susceptible to senescence, along with the passaging, in comparison to MSCs derived from other adult tissues—i.e., bone marrow-derived MSCs. That feature is in favor of their application in more extensive clinical trials [171,172,173].

The terminology used to describe particular cellular populations is unclear and needs to be standardized. Most authors have proposed their own nomenclature based on the origin of the cells. Silini et al. introduced a novel broad definition of perinatal derivatives (PnD), which includes all birth-associated tissues, the cells they are composed of, and all the factors secreted by the mentioned cells along with the conditioned media [174].

### 3.1. Animal Perinatal Mesenchymal Stem/Stromal Cells

The studies conducted on animals paved the way for a better understanding of perinatal MSCs, enabling the first clinical trials in humans. Bailo et al. began the era of perinatal MSCs in modern medicine by isolating and transplanting the human placenta-derived MSCs to swine and rats [175].

Subsequently, many further studies were undertaken to determine the conditions for isolation and to characterize the morphology, immunophenotypes, and other properties of animal perinatal MSCs. Bartholomew et al. confirmed that the collection of umbilical cord tissue and umbilical cord blood for stem cell isolation is a safe procedure for mare and foal pairs in the equine model. They did not observe any changes in time to stand and nurse, nor in hematological parameters in foals or time to pass the placenta for mares [176]. It was found that the equine umbilical cord-derived MSCs are highly proliferative, spindle-shaped cells with the potential to differentiate to osteogenic, chondrogenic, and adipogenic derivatives [177,178,179]. Furthermore, their immunophenotype analyses revealed that they are positive for vimentin, osteonectin, smooth muscle actin, and MHC I and do not express CD31, CD18, MHC II, and the T-cell co-stimulatory molecule CD86 [177].

Shaw et al. examined the amniotic fluid obtained from pregnant ewes in early gestation. They detected the presence of AFMSCs in all of the nine collected specimens (less than 20,000 cells in 10 mL of amniotic fluid). Collected cells were cultured and harvested for up to 20 passages. Cells doubled their count every 36 to 48 h. Analyzed ovine AFMSCs presented the expression of CD44, CD58, and CD166 and were negative for the hematopoietic markers CD14, CD31, and CD45 [180]. Colosimo et al. examined the properties of in vitro cultured ovine AFMSCs. The cells were cultured for 12 passages. They reported that the ovine AFMSCs retained a high proliferation rate up to six passages. However, cells maintained the prolonged expression of CD29, CD58, and CD166 surface molecules, as well as Oct-4, TERT, NANOG, and Sox-2 pluripotency markers (up to 12 passages) [181]. It was discovered that the ovine placental cotyledons are the next readily available source of spindle-shaped, colony-forming, and plastic-adherent ovine perinatal MSCs. Cultured cells showed the features of chondrogenic and osteogenic differentiation. They expressed the CD29, CD44, and CD166 surface markers and were negative for hematopoietic progenitor cell markers [182].

The studies conducted on dogs confirmed that the plastic-adherent perinatal MSCs could be successfully isolated from the canine placental tissue, umbilical cord, and amniotic membrane [183,184,185,186]. All isolated MSCs displayed a fibroblast-like shape in in vitro culture conditions. Saulnier et al. reported that the placenta-derived MSCs exhibited the highest proliferation rate in comparison to other types of perinatal MSCs. They discovered that all cellular populations presented the expression of CD29, CD44, CD73, CD90, CD105, and Sox-2, but did not show the expression of CD34, CD45, MHC II, NANOG, and Oct-4 [185]. The average in vitro canine perinatal MSC population doubling time ranged from 21–42 h and depended on the tissue of their origin [183]. It is postulated that canine MSCs are non-tumorigenic, as Borghesi et al. found no tumor formation features in nude mice after the transplantation of MSCs derived from canine amniotic membranes [184]. Finally, in strictly controlled conditions, all cell populations have the potential to differentiate into adipocytes, osteo-, and chondroblasts [183,184,185].

### 3.2. Human Perinatal Mesenchymal Stem/Stromal Cells

Silini et al. have also proposed the systematized nomenclature and classification of human perinatal tissues and cells. According to their definition, based on their location, the following human perinatal MSCs could be distinguished: human amniotic membrane mesenchymal stromal cells (hAMSC), human placental amniotic membrane mesenchymal stromal cells (hPAMSC), human reflected amniotic membrane mesenchymal stromal cells (hRAMSC), human chorionic mesenchymal stromal cells (hCMSC), human chorionic plate mesenchymal stromal cells (hCP-MSC), human chorionic plate mesenchymal stromal cells derived from blood vessels (hCP-MSC-bv), human chorionic villi mesenchymal stromal cells (hCV-MSC), human chorion leave mesenchymal stromal cells (hCL-MSC). The umbilical cord-derived MSCs: human umbilical cord amniotic mesenchymal stromal cells (hUC-AMSC), human umbilical cord Wharton’s jelly mesenchymal stromal cells (hUC-WJ-MSC), human umbilical cord sub-amnion Wharton’s jelly mesenchymal stromal cells (hUC-saWJ-MSC), and a lineage of human umbilical cord intermediate Wharton’s jelly mesenchymal stromal cells (hUC-iWJ-MSC). Finally, the population of human amniotic fluid mesenchymal stromal cells (hAF-MSC), human basal decidua mesenchymal stromal cells (hBD-MSC), and human parietal decidua mesenchymal stromal cells (hPD-MSC) [174] were also distinguished.

#### 3.2.1. Placenta-Derived Mesenchymal Stem/Stromal Cells

Historically, participants of the First International Workshop on Stem Cells from Placenta (2007) proposed the first systematic classification of human placental stem cells based on their origin and characteristics. They distinguished the following placenta-derived stem cell subpopulations: (1) human amniotic epithelial cells (hAEC), (2) human amniotic mesenchymal stromal cells (hAMSC), (3) human chorionic mesenchymal stromal cells (hCMSC), and (4) human chorionic trophoblastic cells (hCTC) [187]. Those cellular subpopulations emerged from the amniotic and chorionic membrane tissue. The same experts established the minimal criteria for the definitions of hAMSC and hCMCS. Both cell lineages: (1) have a capacity to adhere to plastic in in vitro conditions; (2) form fibroblast colony-forming units; (3) from in vitro passages 2 to 4; are positive for CD90, CD73, and CD105 antigens and do not express the CD45, CD34, CD14, and HLA-DR surface antigens; (4) have the potential to differentiate into one or more lineages, including osteogenic, adipogenic, chondrogenic, and endothelial; and (5) have a fetal origin [187]. Subsequent studies revealed that placenta-derived MSCs could also be isolated from chorionic villi samples [188,189,190,191] and maternal *decidua basalis* [192,193].

**Figure 2:**
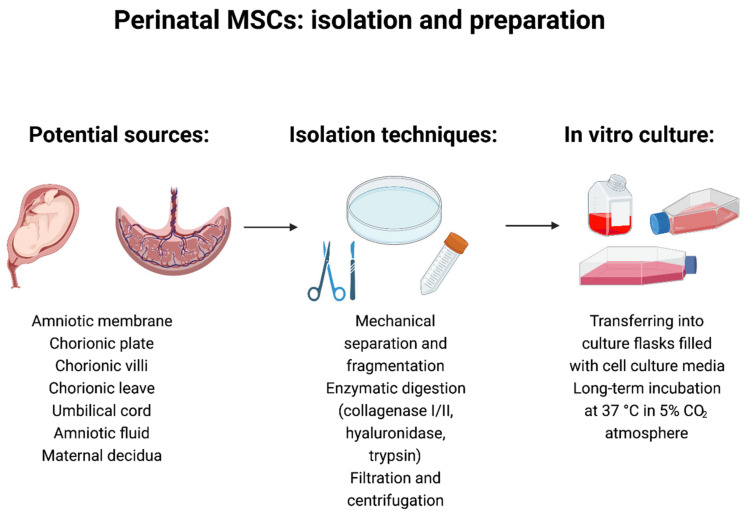
Perinatal mesenchymal stem/stromal cells: isolation and preparation techniques. Created with BioRender.com (accessed on 5 October 2021).

In standard collection protocols, the samples of placental tissue are surgically dissected. The chorionic and amniotic membranes are manually separated and minced into small pieces. Subsequently, cells are isolated from the tissue by the enzymatic digestion (dispase II and collagenase 2). In the next step, the solution is filtered and transferred into culture dishes. Finally, the authors treat isolated cells with various basal culture media supplemented with different fetal bovine serum concentrations (10–20%) and other supplements, such as epidermal growth factor and antibiotics (Figure 2) [194,195,196,197,198].

It has been reported that the hAMSCs have a fibroblast-like cell shape and do not change their morphology in in vitro culture conditions up to five passages. Moreover, they can be easily distinguished from the hAEC because they are three times larger than hAEC [199]. Tested hAMSC cultures reached the senescence after 5–15 passages [199,200,201]. Their immunophenotype analyses revealed the presence of the following antigens: i.a. CD29, CD44, CD73, CD90, CD105, CD166, SSEA-3/4, CK18, HCAM-1, and HLA ABC, and were negative for the CD14, CD34, CD45, TRA-1-60, VCAM-1, PECAM-1, and HLA-DR—shown in Table 1 [199,200,201]. Moreover, they exhibited the expression of the Oct-3/4, GATA-4, Rex-1, BMP-4, SCF, NCAM, nestin, HFN-4alpha, CK18, and vimentin genes, but did not show the expression of BMP2, FGF-5, Pax-6, and telomerase reverse transcriptase [199,200]. To date, no significant differences between the hAMSC obtained from different regions of amniotic membranes have been found [174].

Human chorionic membrane, chorionic plate, chorionic villi, and chorionic leave MSCs have been distinguished due to the place of their origin. However, these cellular populations have similar cellular characteristics and immunophenotypes consistent with classification criteria established by Parolini et al. [187]. Chorionic membrane MSCs present a fibroblast-like morphology and plastic adherence capacity. The comparative analysis revealed some morphological differences between the various types of perinatal MSC. Araujo et al. reported that the chorionic MSCs are smaller than other perinatal MSCs isolated from amniotic membranes, umbilical cord, or the decidua; however, they exhibited similar proliferation capacity until passage 8 [214]. Chorionic membrane MSCs show the expression of CD13, CD29, CD44, CD54, CD73, CD105, CD166 surface markers, and the absence of CD3, CD14, CD34, CD45, and CD31, and are characterized by high cellular plasticity [202]. Chorionic villi MSCs meet the minimal MSC criteria proposed by the International Society for Cellular Therapy—specifically, they show an expression of CD44, CD73, CD90, CD105, and HLA-ABC and lack expression of CD45, CD34, CD19, and HLA-DR surface molecules [36]. Moreover, they are non-immunogenic, meaning they do not express CD14, CD40, CD56, CD80, CD83, CD86, CD275 immune markers. The expression of Sox-2 is the only detected pluripotency feature of CV-MSCs [215]. Perinatal MSCs isolated from the chorionic plate are positive for CD44, CD73, CD90, CD105, and CD166; do not exhibit the expression of CD14, CD19, CD34, CD45, and HLA-DR surface antigens [203,204]; and express the Oct-4, NANOG, and Sox-2 pluripotent stem cell markers [205]. It has been reported that chorionic plate-derived stem cells possess significantly higher migration and proliferation properties compared with other perinatal stem cells [203,205].

It is also postulated that tissue samples obtained from different individuals have specific cellular characteristics. Tai et al. analyzed the differences in properties of placenta-derived MSCs collected from various areas of the placenta—chorionic plate, amniotic membrane, and decidual plate—in five patients. They discovered only a moderate heterogeneity in osteogenic and adipogenic differentiation potentials in samples obtained from different placental regions in enrolled individuals. Similar heterogeneity was observed in the tubulin acetylation measured in different samples [216]. Moreover, they discovered that only the chorionic plate MSCs could decrease the proliferation of peripheral blood mononuclear cells (PBMCs) triggered by the phytohemagglutinin. All cell lineages decreased the proportions of CD3+/CD8-/IFN-γ+ Th1 and CD3+/CD8-/IL17+ Th17 cells and elevated the proportion of Treg in PBMCs [216]. Placenta-derived MSCs regulate trophoblast functioning by promoting increased cell survival and protecting mitochondria from the effects of oxidative stress and, as a result, facilitate trophoblast invasion in humans [217,218].

Decidua MSCs originate from the maternal part of the placenta. They could be isolated from both *decidua basalis* and *decidua parietalis* tissue. It was found that MSCs isolated from term *decidua basalis* are capable of differentiating into three mesenchymal cell lineages [193]. Furthermore, Macias et al. reported that decidua-derived MSCs differentiate into derivatives of all germ layers [192]. Decidua MSCs form a monolayer of plastic adherent, fibroblast-like cells positive for MSC surface markers (CD44, CD90, CD105, CD146, CD166) and HLA-ABC, and negative for hematopoietic and endothelial markers, as well as the following molecules—CD40, CD80, CD83, CD86, and HLA-DR [193].

#### 3.2.2. Umbilical Cord-Derived Mesenchymal Stem/Stromal Cells

MSCs could be isolated from both the umbilical cord blood and umbilical cord tissue [174]. Yang et al. identified the population of plastic-adherent, fibroblast-like, umbilical cord blood-derived MSCs in ~25% of primary cultures. The detected cells were positive for CD13, CD29, CD44, CD73 (SH3, SH4), CD90, and CD105 (SH2) and negative for CD14, CD31, CD34, CD45, CD51/61, CD64, CD106, and HLA-DR. The isolated cells presented features of adipogenic, osteogenic, and chondrogenic differentiation [219]. Nonetheless, the low presence of MSCs in the umbilical cord blood makes their isolation and cultures non-effective for clinical use [219,220,221,222].

Isolation protocols for MSCs derived from umbilical cord tissue are similar to those used to isolate chorionic and amniotic membrane MSCs. At first, the umbilical cord tissue—Wharton’s Jelly—is manually separated from the cord blood vessels and cut into smaller pieces. Then, the collected samples are directly placed in culture flasks (explant cultures) or enzymatically digested (collagenase I/II, hyaluronidase, trypsin). In the next step, the processed solution is filtered or centrifuged and transferred into culture dishes and cultured in various media at 37 °C in a 5% CO_2_ atmosphere [197,223,224,225]. Fortunately, the isolation of MSCs from the umbilical cord tissue is much more efficient. Some authors postulated that the two embryologically different MSCs populations (hUC-AMSC and hUC-WJ-MSC) are present in the umbilical cord tissue. However, the direct connection between the amniotic membrane and the inner connective tissue makes it difficult to isolate the individual cellular subpopulations [174,226]. Cells isolated from Wharton’s Jelly share the features of other perinatal MSCs. Interestingly, two morphologically distinct types of hUC-WJ-MSC (small-sized subpopulation with a flat cell body, large-sized subpopulation) have been visualized in primary cultures. It was found that the subpopulation of small-sized cells displayed higher expression of several surface antigens (CD44, CD73, CD90, and CD105) [206]. Furthermore, those two cellular subpopulations exhibited different cytoplasmic filament profiles (vimentin and cytokeratin filaments) [207]. Other reports indicate that the umbilical cord-derived MSCs show a mostly fibroblastic morphology and are positive for other cellular markers, i.e., C10, CD13, CD29, CD49b-e, CD51, CD146, CD166, and HLA-ABC. In addition, umbilical cord MSCs express the NANOG, Rex-1, and Sox-2 pluripotency gens. However, the expression of Oct-3/4, SSEA-3, SSEA-4, STRO-1, Tra-1-60, and Tra-1-81 is uncertain due to the contrary results of previous studies [208,209,210,211]. Similar to other subpopulations of perinatal MSCs, umbilical cord-derived MSCs possess high plasticity, and, under specific environmental conditions, could differentiate into all germ layer derivatives [227,228,229,230,231].

#### 3.2.3. Amniotic Fluid-Derived Mesenchymal Stem/Stromal Cells

The amniotic fluid is the next rich source of fetal-origin stem cells collected during diagnostic amniocentesis, therapeutic amnioreduction, or cesarean section [232,233,234,235]. Stem cells suspended in the amniotic fluid form a heterogeneous group of cells with distinct properties and cellular characteristics. Similar to placental tissues, amniotic fluid is abundant in multipotent amniotic fluid mesenchymal stem cells that shed from the placenta and umbilical cord. It was shown that these cells are able to differentiate into osteoblastic, bone-forming cells, demonstrated through alkaline phosphatase (ALP) activity and calcium deposition in the extracellular matrix. The MSCs possess the ability to adhere to plastic flasks, which makes them possible to isolate from the second and third trimester amniotic fluid samples in standard culture conditions [236]. In addition, differentiation toward osteoblasts was more efficient on a gelatin scaffold compared to monolayer culture [237]. The experimental observations confirmed the presence of highly proliferative, colony-forming, spindle-shaped cells in amniotic fluid cultures obtained from full-term cesarean sections. Immunophenotype analysis revealed that the hAF-MSC did not express the hematopoietic and endothelial markers (CD45, CD34, CD31), and expressed the MSC markers (CD73, CD90). The expression of CD105 was detectable, but, significantly lower than observed in other MSC lineages. The expression of Oct-4 was significantly increased in the freshly obtained samples in comparison to in vitro cultured cells [212]. Moraghebi et al. have also reprogrammed the term hAF-MSC into the pluripotent stem cell with a similar expression of Oct-4 and NANOG to human embryonic stem cell lines. The reprogrammed cells had the potential to form teratomas and differentiate into hematopoietic and neural cell lineages [212].

Amniotic fluid can be used as a source of cells with higher potency, known as the amniotic fluid stromal cells (AFSCs). AFSCs have the capacity to differentiate into cells of all three embryonic germ layers without forming tumors. That ability places them somewhere between ESCs and MSCs. The plastic adherent AFSCs are isolated by the positive selection for CD117 surface antigen [232,238]. Cloned, CD117-positive cells express CD29, CD44, CD73, CD90, CD105, SSEA-4, Oct-4, and MHC-I molecules, and are negative for MHC-II, CD80, and CD86 antigens. Whereas the first trimester AFSCs could express NANOG, SSEA-3, TRA-1-60, and TRA-1-81, their expression was not detected in the second trimester cells [167,213]. In addition, AFSCs have the potential to be reprogrammed into the induced pluripotent stem cells [232,239]. The regenerative properties of amniotic fluid-derived stem cells mainly depend on the significant paracrine activity of numerous peptides and cytokines released into the surrounding of damaged tissues [240].

## 4. Signaling Pathways Involved in Fetal-Derived MSC Development and Differentiation

All trophoblast lineages are derived from the trophoectoderm cells of the blastocyst [241]. After the implantation in the uterus, trophoectodermal cells become cytotrophoblasts. Human trophoblastic cells can be distinguished into three subpopulations. Both extravillus cytotrophoblasts and syncytiotrophoblasts are derived from the undifferentiated cytotrophoblast cells [242]. Trophoblastic cells were demonstrated to differentiate in vitro from human embryonic stem cells forming embryoid bodies [243]. Bone morphogenetic protein 4 (BMP4), a member of the transforming growth factor β (TGF-β) superfamily, induced such differentiation, as indicated by Xu et al. [244]. Transcriptomic studies revealed that BMP4-treated embryonic stem cells exhibited increased expression of trophoblastic markers, such as CG-α, CG-β (subunits of human chorionic gonadotropin), placental growth factor, glial cells missing 1 (GCM1), the non-classical HLA class I molecule HLA-G1, and CD9. On the contrary, the expression of genes associated with pluripotency, such as POU domain class 5 transcription factor (POU5F1) or telomerase reverse transcriptase (TERT), was decreased [244].

Cytotrophoblast proliferation is associated with hypoxic conditions (2% O_2_), as indicated by in vitro studies, however, low levels of oxygen do not induce syncytialization [245]. Effects exerted by hypoxia are mediated by the hypoxia inducible factor 1 (HIF-1), which is regulated by the tumor suppressor protein von Hippel-Lindau (VHL) via complex formation. Under normoxic conditions, HIF-1/VHL complex is degraded [242]. Apart from that, hypoxia induces expression of several genes, such as cyclin B1, focal adhesion kinase (FAK), α5 β1 integrin, p53, BAX, TGF-β, or MMP-2 [246].

Syncytialization is a process occurring at implantation when cytotrophoblast cells fuse, which may be influenced by various factors, as indicated in in vitro studies, such as epidermal growth factor (EGF), granulocyte-macrophage stimulating factor (GM-CSF), human chorionic gonadotropin (hCG), glucocorticoids, or estradiol. Syncytin, encoded by an envelope gene of a defective endogenous human retrovirus, HERV-W, induces cell fusion and syncytiotrophoblast formation, as indicated by in vitro studies by Frendo et al. [247]. Connexin 43 was demonstrated to participate in syncytiotrophoblast formation as well [248].

Extravillus cytotrophoblasts comprise several subtypes based on their location, such as cytotrophoblasts of cell columns, interstitial cytotrophoblasts, or endovascular cytotrophoblasts. Differentiation towards extravillus cytotrophoblasts occurs along the invasive pathway, where the cells invade the endometrium [242].

Transcriptomic studies performed by Okae et al. [249] revealed that the genes related to the wingless/integrated (Wnt) and epidermal growth factor (EGF) signaling pathways were overexpressed in cytotrophoblast cells isolated from first-trimester placentas. This led to the establishment of proliferative human cytotrophoblast cells in in vitro culture via activation of Wnt and EGF and inhibition of TGF-β, histone deacetylase (HDAC), and Rho-associated protein kinase (ROCK). Such cultured cells were able to give rise to the three major trophoblastic lineages and, therefore, were designated ‘trophoblast stem cells’. Their differentiation towards extravillus cytotrophoblast cells was dependent on the addition of neuregulin 1 (NRG1), A83-01 (an TGF-β inhibitor), and Matrigel^®^ to the culture, which resulted in epithelial-mesenchymal transition and expression of HLA-G. Trophoblast stem cells were also successfully differentiated towards syncytiotrophoblast and the addition of forskolin (cyclic AMP agonist), EGF, and 3D culture conditions were vital for this purpose [249]. In contrast, the derivation of mouse trophoblast stem cells is dependent on activation of FGF and TGF-β and inhibition of Wnt and ROCK [250].

The development of the human umbilical cord starts after the implantation of the blastocyst. Initially, the embryo is connected to endometrium through the trophoblast, which develops into the connecting stalk, constituting the earliest sign of the umbilical cord [22,251]. The umbilical cord’s connective tissue originates from the extraembryonic mesoblast. Between 28 and 40 days post coitum, the expanding amniotic cavity compresses the connecting stalk, the allantois, and the yolk sac and covers them with the amniotic epithelium, forming the cord. Fetal blood vessels originate from the allantois around the third week post coitum and subsequently develop into umbilical vessels [251].

**Figure 3:**
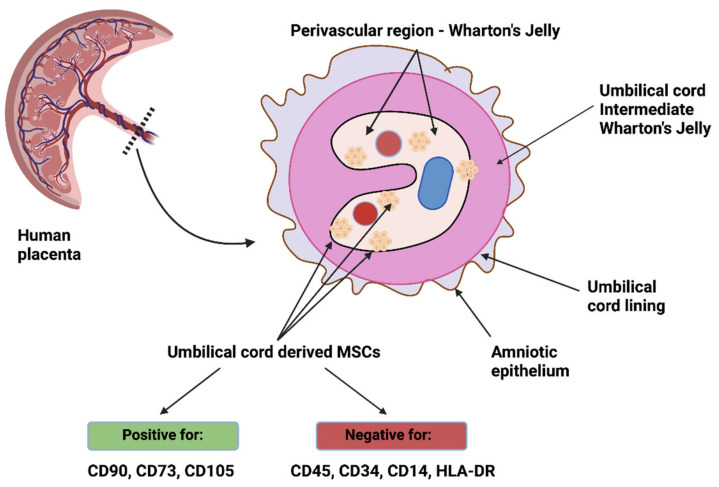
Umbilical cord-derived MSCs—location and characteristics. Created with BioRender.com (accessed on 5 October 2021).

Although stem cells are located in various compartments of the umbilical cord, the stromal tissue, called Wharton’s jelly, provides cells that are the richest in stemness properties [252]. Moreover, these cells are mostly located in the proximity of umbilical vessels in Wharton’s jelly, therefore, it seems that the perivascular region is a source of precursor cells, Figure 3 [253,254].

This hypothesis, related to Wharton’s jelly MSCs, is consistent with the results obtained by Crisan et al. [255]. The authors aimed to investigate the presence of multilineage progenitors among perivascular cells, mostly pericytes, and isolated them from skeletal muscle, pancreas, adipose tissue, placenta, umbilical cord, and other tissues. These cells expressed NG2 (neural/glial antigen 2), CD146, and PDGFRβ (platelet-derived growth factor receptor β), while not expressing endothelial cell markers. When cultured over a prolonged time period, perivascular cells exhibited expression of markers typical for MSCs, such as CD44, CD73, CD90, and CD105, and were able to differentiate towards chondrocytes, adipocytes, and osteocytes, suggesting that MSCs are derived from perivascular cells [255]. Sarugaser et al. [256] isolated a nonhematopoietic human umbilical cord perivascular cell population capable of bone nodule formation and expressing markers typical of MSCs. Interestingly, umbilical cord perivascular cells are also PDGFRβ+ and may be recruited through the PDGFB signaling pathway, especially since amniotic fluid has been demonstrated to contain both PDGFA and PDGFB, which could influence their migration from the vasculature [257,258]. Takashima et al. [259] demonstrated that the earliest wave of PDGFRα+ MSCs in the embryonic trunk was generated from Sox1+ neuroepithelium partially through a neural crest pathway. Nevertheless, MSCs may also be isolated from non-perivascular regions of the umbilical cord, such as the umbilical cord lining, however, they could have simply migrated away from the vasculature [260].

Moreover, Wharton’s jelly-derived MSCs exhibit properties of both fibroblasts (the expression of vimentin) and smooth muscle cells (the expression of desmin, actin, and myosin) [253] and, therefore, are regarded as myofibroblasts. As indicated by Nanaev et al. [21], the level of differentiation of stromal cells towards myofibroblasts is dependent on the stage of gestation, and the most differentiated cells are located in the perivascular zone of Wharton’s jelly.

The amnion adheres to the umbilical cord and fetal skin and is extended from the edge of the placenta. This fetal membrane is composed of an epithelial monolayer contacting the amniotic fluid and four layers of connective tissue of mesodermal origin. The connective tissue layer is composed of fibroblast-like mesenchymal cells and a collagenous extracellular matrix. Importantly, the amniotic membrane regulates the volume and composition of amniotic fluid, which consists of amniotic fluid-derived MSCs [261]. However, the cellular component of the amniotic fluid changes during gestation, receiving cells shed from the fetus or possibly containing cells derived from the placenta or the inner cell mass of the morula [262]. It has also been hypothesized that embryonic cell mass releases a variety of stem cell types into the amniotic cavity, which are transported by the amniotic fluid and implant various tissues [263].

Torricelli et al. [264] obtained small nucleated round cells from the amniotic fluid before the 12th week of gestation, which were identified as hematopoietic progenitor cells originating from the yolk sac. This is consistent with the results of Pieternella et al. [265], who demonstrated that amniotic fluid-derived cells are of fetal origin, as indicated by molecular HLA typing. Moreover, these cells exhibited properties of MSCs, such as multilineage differentiation potential towards fibroblasts, adipocytes, osteocytes [265], and chondrogenic lineage [266]. Ovine mesenchymal amniocytes were shown to give rise to smooth and skeletal muscle cells after the treatment with promyogenic medium, which resulted in expression of transgelin, calponin, and α-actin [267].

However, recently, third-trimester amniotic fluid was demonstrated to contain MSCs of renal origin [268]. These cells were not only positive for pluripotency markers, such as SSEA-4 (stage-specific embryonic antigen 4), c-kit, or TRA-1-60, but also expressed the master renal progenitor markers: SIX2 (SIX homeobox 2) and CITED1 (CBP/p300-interacting transactivator 1), as well as renal proteins, including PODXL (podocalyxin like), LHX1 (LIM homeobox 1), BRN1 (POU class 3 homeobox 3), and PAX8. Moreover, these cells exhibited renal functions, as demonstrated by albumin endocytosis assays, and gene ontology terms revealed their involvement in pathways associated with kidney morphogenesis [268].

Both renal and osteoblastic differentiation was reported to be dependent on mTOR (mechanistic target of rapamycin) signaling cascade. Importantly, this pathway was demonstrated to be fully active in amniotic fluid stem cells by Siegel et al. [269]. Blocking intercellular activity of mTOR via the inhibitor rapamycin or through siRNA resulted in diminished embryonic body formation by amniotic fluid stem cells, which constitutes the principal step in differentiation of pluripotent embryonic stem cells. Specifically, embryonic body formation was reported to be dependent on two complexes, namely mTORC1, which regulates mRNA translation via kinase phosphorylation, and mTORC2, which phosphorylates and subsequently activates AKT [270].

## 5. Animal Models and Clinical Applications

Human and animal stem cells of fetal origin have been the subjects of numerous studies that aimed to find their possible applications in daily clinical practice. The first reports about the unique properties of cells derived from human amniotic membranes were released almost two decades ago. Balio et al. described a breakthrough in the field of perinatal MSC transplantation. They successfully transplanted the human amnion and chorion cells, obtained from term placentas, to neonatal swine and rats. They discovered that the obtained cells did not induce allogeneic or xenogeneic lymphocyte proliferation [175], making them viable therapeutic candidates.

### 5.1. Animal Models

Laboratory animals have been used in medical research for many years. Veterinary medicine provides a tool to study transplantation mechanisms between basic science and clinical human medicine. Most human diseases also affect animals, hence the etiopathogenesis and treatment are similar. Veterinary medicine, thus, represents a valuable field, especially in regenerative medicine, where the development of animal-based protocols could be transferred to human medicine. A key issue is the collaboration of researchers from different fields, including physicians, veterinarians, biologists, geneticists, and others, and working together in accordance with the “One Health” mindset [271].

#### 5.1.1. Bone and Cartilage Diseases

With regard to bone repair processes, SCID mice were used that were subcutaneously implanted with MSCs from human placental chorion and MSCs from the *decidua basalis* [272]. In both cases, ectopic bone formation was observed at 8 weeks. The expression of the markers osteopontin (OPN), osteocalcin (OCN), biglikan (BGN), and bone sialoprotein (BSP), which are characteristic of bone tissue, was also demonstrated. Placenta-derived MSCs are, therefore, cells with bone-forming potential [272]. Similar findings were presented in a Wistar rat model, where, among MSCs from different sources (bone marrow, Wharton’s jelly, umbilical cord, placenta, adipose tissue), human placental MSCs showed the highest osteogenic potential and complete bone regeneration [273].

Human amniotic MSCs were differentiated into chondrocytes, as confirmed by the expression of SOXs and BMPs, and then transplanted into non-cartilage tissues in mice and on a collagen scaffold into defects in rat bone. Morphological changes to the transplanted MSCs, along with deposition of type II collagen, were observed, suggesting their potential use in the treatment of osteoarthritis [274]. A rabbit model was also used to study the treatment of cartilage damage, where placental MSCs were applied to a silk fibroin biomaterial scaffold [275]. Damaged femoral condyles lacking articular cartilage were analyzed after implantation of MSCs. Defect repair occurred within 4–12 weeks and no more degeneration or inflammatory cell infiltration was observed thereafter. Another study using a rabbit model for cartilage damage in the knee joint showed similar results [276]. MSCs from amniotic fluid were differentiated on chondrogenic medium with TGFβ3 and BMP2 and xenotransplantation allowed in vivo survival for 8 weeks [276].

#### 5.1.2. Cardiac Diseases

Due to the fact that adult cardiomyocytes do not regenerate after injury, treatment of heart failure continues to pose problems. Zhao et al. [277] investigated the feasibility of using human amniotic MSCs to treat heart injury in a rat model. MSCs were cultured with neonatal rat heart explants and then transplanted into infarcted rat hearts. During culture, MSCs were stimulated with bFGF and activin A and shown to express Nkx2.5 and atrial natriuretic peptide (specific for cardiomyocytes). The cardiac-specific myosin alpha heavy chain gene was also detected. When cultured together with explants, MSCs integrated and differentiated into the host tissue. After transplantation, MSCs were maintained for 2 months as similar to cardiomyocytes [277]. Animal models have also been used to study the effects of MSCs on myocardial infarction healing [278,279]. MSCs from Wharton’s jelly were transplanted into mouse [279] and mini-swine [278] models. Both studies demonstrated decreased apoptosis in injured myocardium, cardioprotective effects, and increased capillary density. Nevertheless, studies in the mini-swine model demonstrated differentiation of MSCs into cardiomyocytes and endothelial cells. Human placenta-derived MSCs were also used to study myocardial infarction in a porcine model. MSCs were preconditioned with hyaluronan mixed with butyric and retinoic acid ester. Implantation of these MSCs reduced scar size and increased capillary density and myocardial perfusion, along with a reduction in fibrous tissue [280].

#### 5.1.3. Neurological Disorders

MSCs isolated from Wharton’s jelly have been used to study Parkinson’s disease using a rat model. The animals were induced to have forebrain lesions, causing movement disorders. A reduction in motor deficits was observed in rats after MSC administration, explained by the protection of dopaminergic neurons by growth and neurotrophic factors secreted by MSCs [281,282]. Human placenta-derived MSCs were also used in studies of Parkinson’s disease treatment in a rat model [283]. Almost normal motor function was observed 24 weeks after MSC transplantation. Through immunohistochemical and positron emission tomography (PET) analyses, dopaminergic differentiation of progenitors was demonstrated, indicating that neuronal progenitors can differentiate eventually in vivo and alleviate motor defects [283]. A mouse model of Alzheimer’s disease (AD) was used to study the effects of MSCs from human placenta [284]. These cells were given to mice intravenously and the first effects observed were improved spatial learning ability correlated with fewer Aβ plaques in the brain. There was also a decrease in pro-inflammatory and an increase in anti-inflammatory cytokines in mice after MSC administration compared to animals receiving saline. Improvement of AD pathology through paracrine processes and immune modulation was indicated [284].

A rat model was also recently used to study the anti-inflammatory effects of Wharton’s jelly MSCs in spinal cord injury [285]. The study used real-time polymerase chain reaction, Western blotting, and ELISA and determined the expression levels of NLRP1, ASC, active caspase-1, interleukin-1beta (IL-1β) and IL-18, and TNF-α. These factors are responsible for the local inflammatory response. The results indicated decreased expression in rats with injured spinal cords that received MSC transplantation and, in addition, the motor functions of the animals were improved [285]. Interesting studies have also been conducted in rodent models for the treatment of spinal cord injury using umbilical cord MSCs. The spleen was shown to be an important organ mediating the effects of MSCs, through their immunomodulatory action, stimulating, for example, specific inflammatory cytokines that recruit immune cells. Splenectomized animals lost the ability to reduce spinal cord hemorrhage and did not increase systemic IL-10 levels after MSC administration [286]. In contrast, another study, also in a rat model with a damaged cord, compared the effects of MSCs alone with Wharton’s jelly and conditioned medium [287]. Cells in culture exhibit paracrine activity, hence the potential action of factors contained in conditioned medium (CM). Rats with compressive lesions were administered MSCs and CM intrathecally. In both cases, improvement and increased expression of genes related to axonal growth were observed. However, when MSCs were used, expression of inflammatory markers was demonstrated, which is a result of the inflammatory response to the transplant. In the case of CM, no inflammatory response was shown, and, in addition, axonal sprouting was improved and the number of reactive astrocytes decreased [287]. Similarly, treatment of spinal cord injury in a rat model was studied using Wharton’s jelly MSCs. In the study described here, MSCs were administered intrathecally at different concentrations and repetitions. The results showed a positive but dose-dependent effect of MSCs on spinal cord regeneration [288].

The experimental animals modeling multiple sclerosis were administered human placenta-derived MSCs (hPMSCs) and low and high doses of extracellular vesicles from hPMSCs (hPMSCs-EVs), as well as saline. High doses of hPMSCs-EVs resulted in improved motor function. Both hPMSCs-EVs and hPMSCs reduced DNA damage in oligodendroglia and also increased myelination in the spinal cord [289]. Research on the use of MSCs to treat multiple sclerosis has also been conducted previously in a mouse model [290]. Human placental MSCs (hPMSCs) were administered intracerebrally to mice with experimental autoimmune encephalomyelitis in an MS model. Both survival and reduced disease severity were observed, and the effects were attributed to a reduction in the anti-inflammatory protein TSG-6 [290].

The rat model has also been used to study MSC therapy in neural tissue ischemia. MSCs from Wharton’s jelly were transplanted into rats intracerebrally and were shown to differentiate into glial cells and neuronal cells and also showed increased angiogenesis. The regulation of beta1-integrin was shown to include an important role in the processes that promote plasticity of MSCs when transplanted intracerebrally [150]. The effect of human umbilical cord-derived stem cells on the treatment of neonatal hypoxic–ischemic encephalopathy was also studied in a rat model [33]. Motor and cognitive functions were shown to be improved and caspase-3 and Beclin-2 expression was decreased, suggesting the potential for MSCs in the treatment of hypoxic–ischemic encephalopathy.

#### 5.1.4. Organ Disorders

The rat and mouse model was also used to study the effect of MSCs from Wharton’s jelly on liver [291] and lung [292] fibrosis. In both cases, the amount of collagen was reduced, which improved organ function, and inflammation was also shown to be reduced. Similar studies were also conducted later using intravenously administered human placental MSCs with green fluorescent protein (GFP) expression in the treatment of liver fibrosis in a rat model [293]. Alleviation of liver fibrosis, reduction of collagen area, reduction of TGF-β1 and α-SMA (markers of fibrosis) expression and improvement of rat organ function were obtained. Liver regeneration by MSCs was examined using a rat model with carbon-tetrachloride-damaged liver tissue [294]. Human MSCs derived from chorionic platelets were used in this study. The expression of markers related to autophagy, apoptosis, cell survival, and liver regeneration were analyzed. The results indicated that MSCs induced tissue repair through HIF-1α-mediated mechanisms and autophagy [294]. The effect of human MSCs from amniotic membrane on CCl_4_-induced cirrhosis in mice was studied [295]. The isolated MSCs were injected into the spleens of mice, which were then sacrificed after 4 weeks. Alanine aminotransferase (ALT) and aspartate aminotransferase (AST) levels in the blood of mice were evaluated and histological analysis of the liver was performed. Reduced areas of liver fibrosis and improved blood parameters (ALT, AST) were observed in mice after MSC injection compared to the control group. Activation of hepatic stellate cells and apoptosis of hepatocytes were decreased, while regenerative processes were promoted. The injected MSCs showed expression of hepatocyte-specific markers (α-fetoproteinran and human albumin) [295]. Due to the high genetic similarity of pigs to humans, as well as similar organ size, clinical studies on a porcine model are particularly valuable. The study by Cao et al. [296] used Chinese miniature pigs with acute liver failure that were implanted with human placental MSCs. Histological analysis showed a reduction in liver inflammation, liver denaturation, and necrosis and also showed promotion of liver regeneration; however, these observations applied only to the group of pigs injected with MSCs via the portal vein. Those that received administration of MSCs through the jugular vein or treatment of MSCs with X-rays prior to injection and the control group did not show these changes. The authors suggested that the portal vein infusion route with the help of B-ultrasound is more favorable compared to the jugular vein [296].

The isolated MSCs from cord blood were used to study colitis in a mouse model [297,298]. The first study used NOD2-activated MSCs from cord blood that were injected intraperitoneally. It was shown that MSCs inhibited the inflammatory response and activated the anti-inflammatory response in the colon [297]. In turn, the second study analyzed extracts from MSCs injected intraperitoneally into mice. It was shown that the extracts strongly inhibited the inflammatory process and increased the body weight of the animals. A shift in the functional phenotype of macrophages from M1 to M2 was observed [298].

The mouse model was also used to study the effect of MSCs from human amniotic fluid on the treatment of stress urinary incontinence [299]. MSCs were first differentiated in vitro for the myogenic direction, as confirmed by the expression of PAX7, MYOD, and dystrophin. MSCs were labeled with silica-coated magnetic nanoparticles containing rhodamine B isothiocyanate and then injected transurethrally into mice with gluteal nerve injury. Nerve regeneration and neuromuscular junction formation were demonstrated by expression of neuronal markers and acetylcholine receptor. Transurethral injection of MSCs resulted in a return to normal histological structure of the urethral sphincter and also a definite improvement in function, while lacking tumorigenicity and immunogenicity [299].

#### 5.1.5. Ischemia and Wound Healing

MSCs isolated from the umbilical cord were tested in a mouse model of hind limb ischemia. MSCs were isolated and cultured in vitro and then differentiated in endothelial differentiation medium with VEGF and bFGF. Transplanted cells into the mouse hind limb differentiated into endothelial cells, indicating the potential use of these cells in promoting angiogenesis and reendothelialization [230].

Animal models involving mice were used to study wound healing using Wharton’s jelly MSCs that were placed on a scaffold decellularized from amniotic membrane. Results showed accelerated wound healing, reduced scarring, and also hair growth on the treated skin [300].

### 5.2. The Application of Perinatal MSC in Human Clinical Trials

The PubMed database was searched for the relevant references from the last five years until April 2021 to summarize the latest reports on the perinatal MSC application in the completed clinical trials. We searched the PubMed database using the following terms: “placenta mesenchymal stem cells”, “umbilical cord mesenchymal stem cells”, and “amniotic fluid stem cells”. We set the article type to “Clinical Trial” and the species to “Humans” for additional searching filters.

#### 5.2.1. Placenta-Derived MSCs

Regenerative medicine opens novel perspectives for the clinical application of placenta-derived MSCs. Soltani et al. analyzed the safety and efficacy of intra-articular injection of placenta-derived MSCs in knee osteoarthritis treatment. Patients did not present any acute and long-term severe adverse effects of MSC therapy. The first results were promising—eight weeks after the procedure, a significant increase in knee flexion range of motion and pain reduction was noted. Patients also reported a significant improvement in their daily activity and quality of life. However, they did not observe any significant long-term clinical improvements and chondral regeneration (24 weeks after the procedure) [301]. The list of the most recent human perinatal MSC clinical trials is shown in Table 2. Winkler et al. investigated the profile of safety and the potential dose-dependent benefits from the intramuscular injection of placenta-derived MSCs in the patients who underwent the hip arthroplasty that was used as a standardized injury model. The MSC administration was well tolerated and did not cause any adverse effects. Interestingly, the patients who received the lower dose of placenta-derived MSCs had better post-operative results. They had significantly improved muscle strength and volume compared with placebo. Furthermore, the administration of placenta-derived MSCs reduced the levels of surgery-related inflammatory biomarkers [302]. Norgren et al. published the protocol of their planned clinical trial that aims to assess the safety and the efficiency of placenta-derived MSC intramuscular injections performed in patients with atherosclerotic critical limb ischemia who are unsuitable for the standard revascularization [303]. Levy et al. reported that the intrapenile placenta-derived MSC injection increased the penile peak systolic velocity at 3 and 6 months after the procedure. At 3 months after the procedure, 3 out of 8 patients were able to achieve erection [304]. Finally, Zeng et al. described a successful single-case report of placenta-derived MSC hydrogel application to treat the diabetic foot ulcer [305].

Immunomodulatory properties of placenta-derived MSCs could be applied to the treatment of chronic inflammatory diseases. Haller et al. performed the new drug’s clinical trial for patients with chronic obstructive pulmonary disease. The drug contains a mixture of anti-inflammatory molecules obtained from the exosomes isolated from the placenta-derived MSCs. After three (one per week) inhalations with the new drug, treated patients were found to have significantly increased spirometry parameters (FEV1, PEF) and improvements in CT pulmonary images [306]. Hashemian et al. focused on the possible implementation of the perinatal MSC transplantations in the treatment of patients with COVID-19-induced ARDS. They discovered that the cell therapy was safe and could be associated with reduced dyspnea and increased SpO_2_ within 48–96 h after the intervention [307]. Ringden et al., in their non-randomized trial, reported that the decidua-derived MSCs could be used as a promising new tool for the treatment of severe acute graft-versus-host disease [308].

#### 5.2.2. Umbilical Cord and Amniotic Fluid-Derived MSCs

Similar to placenta-derived MSCs, umbilical MSCs have found numerous applications in regenerative medicine. Several clinical trials focused on their utility in the treatment of osteoarthritis. Patients treated with MSCs via intra-articular injections experienced a significant decrease in knee pain and improvements in knee joint function and clinical parameters, such as Western Ontario and Mc Master Arthritis Indexes. The MSC therapy was more efficient than the hyaluronic acid injections—its effect was stable over seven years of follow-up [309,310,311,312]. He et al. analyzed the efficacy of intramyocardial grafting of collagen scaffolds covered with umbilical cord-derived MSCs in patients with chronic ischemic heart disease. They did not observe any significant benefits (reduction of the infarct size) from the cellular therapy compared with the control group [313]. Interestingly, it was reported the umbilical cord MSC infusion was connected with the significant improvement in the left ventricular ejection fraction in patients with heart failure [314]. Hashemi et al. noted that the human umbilical cord-derived MSCs seeded on the acellular amniotic membrane scaffolds improved the process of healing of chronic skin ulcers in patients with diabetes [315]. In addition, the umbilical cord-derived MSC conditioned media could be used in the novel protocols of regenerative medicine. Kim et al. reported that the topical drugs with umbilical cord-derived MSC conditioned media improved the skin stratum corneum and strengthened the skin barrier in patients with atopic dermatitis [316]. Finally, it was found that the application of umbilical cord blood-derived MSC conditioned media containing cream or serum reduced the total area of microcrusts and erythema in patients treated with ablative CO_2_ fractional laser [317].

The immunomodulatory properties of the umbilical cord-derived MSCs may successfully modify the course of chronic inflammatory and degenerative diseases. Therefore, several studies have aimed to investigate their feasibility and efficacy in the treatment of rheumatoid arthritis. MSC therapy was found to be safe and associated with reduced levels of inflammatory parameters and disease activity [318,319,320,321]. Other factors, like cervus and cucumis peptides and interferon-γ combined with MSCs, could improve the therapy effects [318,319]. Large analysis revealed that the umbilical cord-derived MSC transplantations are safe and do not cause serious adverse effects in patients with chronic autoimmune rheumatoid diseases, such as systemic lupus erythematosus, Sjögren’s syndrome, and systemic sclerosis. However, Deng et al. declared that the MSC therapy has no significant positive effects in patients with lupus nephritis [322]. Riordan et al. reported no serious adverse effects of MSCs therapy in patients with multiple sclerosis. Assessment performed one month after the treatment revealed that the treated patients experienced positive changes in the bladder, bowel, non-dominant hand, and sexual functions, as well as in the results of walk tests and general quality of life [323]. Allogenic umbilical cord-derived MSCs were also used to treat individuals with cerebral palsy and autism spectrum disorders. The therapy combined with rehabilitation improved the gross motor and comprehensive function in children with cerebral palsy, and was associated with lower levels of inflammatory markers and better clinical outcomes in patients with autism disorders [324,325].

Moreover, allogeneic umbilical cord-derived MSC infusions were found to be safe in patients with Crohn’s disease. The randomized controlled trial outcomes revealed that patients treated with MSCs had significantly reduced disease activity index and corticosteroid dosage, compared with the controls, at 12 months after the cell therapy [326]. Some authors speculated that the unique immunomodulatory properties of umbilical MSCs could modulate the immune response in allogeneic graft recipients. MSCs were administered as supplementary induction therapy in patients who underwent kidney transplantation. The applied procedure was found to be safe for enrolled patients. However, there was no evidence of its efficacy in comparison with the control group treated with standard immunosuppressive drugs [327]. However Shi et al. discovered that umbilical cord-derived MSC modifies the course of acute liver allograft rejection. Their analyses revealed that the MSCs transplantation increased the T-regulatory/T-helper 17 cells ratio, TGF-β1, and prostaglandin E2 levels [328].

It was discovered that the umbilical cord-derived MSC infusions are safe and well-tolerated in patients with severe COVID-19 infection. The results of the first clinical trials indicate that the MSCs therapy could be potentially efficient in the management of COVID-19 cases. The authors observed several improvements in the treated patients compared with the placebo—in the CT imaging outcomes and clinical parameters, such as the results of 6 min walk tests. However, further large phase III clinical trials are needed to confirm those mostly positive tendencies [307,329,330,331]. He et al. administered a single infusion of umbilical cord-derived MSCs in patients with severe sepsis. They reported that the experimental procedure was safe and well-tolerated. Nonetheless, to investigate its efficacy in that indication, further studies are still needed [332].

Even though the animal models brought us the first promising outcomes, there have been no studies that assessed the safety and efficacy of amniotic fluid-derived stem cells in experimental treatment protocols in humans [232]. It was demonstrated that after the appropriate preparation, the population of amniotic fluid-derived MSCs could be used as a model in the studies focused on human genetic diseases. Squillaro et al. reported that they silenced glucocerebrosidase (GBA) and alpha-galactosidase A (GLA) genes in the amniotic fluid-derived MSCs, creating the models of Gaucher and Fabry diseases. They found that GBA and GLA silencing provoked impaired autophagy and DNA repair mechanisms and, as a consequence, promoted apoptosis and increased senescence in the investigated cellular populations. Moreover, the authors concluded that the perinatal cells collected from fetuses affected by genetic diseases could be used as a readily available source of experimental materials alternative to animal models [333].

Multiple phase I/II clinical trials confirmed the safety of perinatal MSC administration in the regenerative medicine protocols and the treatment of various chronic diseases. However, their efficacy should be investigated in further large randomized controlled trials.

**Table 2:** Perinatal MSCs in human clinical trials.

Condition/ Procedures	Type of Study	Number of Participants	Material	First Author; Year; Reference
Knee osteoarthritis	Randomized, double-blind, placebo-controlled clinical trial	20	Allogenic placenta-derived MSCs	Soltani; 2019; [301]
Hip arthroplasty	Randomized, double blind, placebo-controlled, phase I/IIa clinical trial	20	Allogenic placenta-derived MSCs	Winkler; 2018; [302]
Erectile dysfunction	Prospective, non-randomized, single-arm clinical trial	8	Allogenic placenta-derived MSCs	Levy; 2016; [304]
Chronic obstructive pulmonary disease	Prospective, non-randomized, single-arm clinical trial	30	Allogenic placenta-derived MSCs-derived product: Exo-d-MAPPS drug	Harrell; 2020; [306]
COVID-19-induced ARDS	Prospective, non-randomized, single-arm clinical trial	11	Allogenic placenta-derived MSCs (5 cases) Umbilical cord-derived MSCs (6 cases)	Hashemian; 2021; [307]
Acute graft-versus-host disease	Prospective, non-randomized, single-arm clinical trial	38	Allogenic decidua-derived MSCs	Ringden; 2018; [308]
Knee osteoarthritis	Single-arm, open-label clinical trial	29	Allogenic umbilical cord-derived MSCs	Dilogo; 2020; [309]
Knee osteoarthritis	Randomized, placebo-controlled, phase I/II clinical trial	26	Allogenic umbilical cord-derived MSCs	Matas; 2019; [310]
Knee osteoarthritis	Open-label, single-arm, single-center, phase I/II clinical trial with 7-year extended follow-up	7	Allogeneic human umbilical cord blood-derived MSCs	Park; 2017; [311]
Knee osteoarthritis	Randomized, placebo-controlled clinical trial	36	Allogenic umbilical cord-derived MSCs	Wang; 2016; [312]
Chronic ischemic heart disease	Randomized, double-blind clinical trial	115	Collagen scaffolds covered with umbilical cord-derived MSCs	He; 2020; [313]
Heart failure	Randomized, controlled, phase I/II clinical trial	30	Allogenic umbilical cord-derived MSCs	Bartolucci; 2017; [314]
Chronic diabetic skin ulcers	Randomized, clinical trial	5	Allogenic umbilical cord-derived MSCs seeded on biological scaffold	Hashemi; 2019; [315]
Atopic dermatitis	Prospective, non-randomized, single-arm clinical trial	28	Topical drugs with allogenic umbilical cord-derived MSCs conditioned media	Kim; 2020; [316]
Ablative CO_2_ fractional laser treatment	Randomized, double-blinded, controlled clinical trial	23	Umbilical cord blood-derived MSCs conditioned media containing serum	Kim; 2020; [317]
Rheumatoid arthritis	Randomized, controlled clinical trial	119	Allogenic umbilical cord-derived MSCs	Qi; 2020; [319]
Rheumatoid arthritis	Randomized, controlled, phase I/II clinical trial	63	Allogenic umbilical cord-derived MSCs	He; 2020; [318]
Rheumatoid arthritis	Prospective, phase I/II clinical trial	64	Allogenic umbilical cord-derived MSCs	Wang; 2019; [320]
Rheumatoid arthritis	Open-label, single-arm, single-center, phase Ia clinical trial	9	Allogenic umbilical cord blood-derived MSCs	Park; 2018; [321]
Lupus nephritis	Randomized, double-blind, placebo-controlled clinical trial	18	Allogenic umbilical cord blood-derived MSCs	Deng; 2017; [322]
Multiple sclerosis	Prospective, non-randomized, single-arm clinical trial	20	Allogenic umbilical cord-derived MSCs	Riordan; 2018; [323]
Cerebral palsy	Randomized, controlled clinical trial	39	Allogenic umbilical cord-derived MSCs	Gu; 2020; [324]
Autism spectrum disorder	Single-arm, phase I/II clinical trial	20	Allogenic umbilical cord-derived MSCs	Riordan; 2019; [325]
Crohn’s disease	Randomized, controlled clinical trial	82	Allogenic umbilical cord-derived MSCs	Zhang; 2018; [326]
Kidney transplantation	Multicenter, randomized, controlled clinical trial	42	Allogenic umbilical cord-derived MSCs	Sun; 2018; [327]
Acute liver allograft rejection	Randomized, controlled, clinical trial	27	Allogenic umbilical cord-derived MSCs	Shi; 2017; [328]
COVID-19	Randomized, double-blind, placebo-controlled, phase II clinical trial	101	Allogenic umbilical cord-derived MSCs	Shi; 2021; [329]
COVID-19	Parallel assigned controlled, non-randomized, phase I clinical trial	18	Allogenic umbilical cord-derived MSCs	Meng; 2020; [330]
COVID-19	Single-center open-label, individually randomized, standard treatment-controlled clinical trial	41	Allogenic umbilical cord-derived MSCs	Shu; 2020; [331]
Sepsis	Single-center, open-label, dose-escalation phase 1 clinical trial	15	Allogenic umbilical cord-derived MSCs	He; 2018; [332]

## 6. Conclusions

Due to the indicated properties of differentiation into other cell types, MSCs show great potential for application in regenerative medicine. Additionally, the simplicity of obtaining cells from perinatal tissues during routine procedures and minimal ethical concerns make perinatal tissues one of the most valuable sources for MSCs. Promising results from clinical trials indicate possible therapeutic use. It seems, therefore, necessary to describe a universal and simple isolation protocol for the standard use of MSCs in medicine.

## Figures and Tables

**Table 1:** Expression of cellular markers in various populations of perinatal mesenchymal stem/stromal cells (MSCs).

Cells	Positive Expression (+)	Negative Expression (−)	References
Amniotic MSCs	CD29, CD44, CD73, CD90, CD105, CD166, SSEA-3/4, CK18, HCAM-1, HLA ABC, Oct-3/4, GATA-4, Rex-1, BMP-4, SCF, NCAM, nestin, HFN-4alpha, CK18, vimentin	CD14, CD34, CD45, TRA-1-60, VCAM-1, PECAM-1, HLA-DR, BMP2, FGF-5, Pax-6, TERT	[199,200,201]
Chorionic membrane MSCs	CD13, CD29, CD44, CD54, CD73, CD105, CD166	CD3, CD14, CD34, CD45, CD31	[202]
Chorionic villi MSCs	CD44, CD73, CD90, CD105, HLA-ABC, Sox-2	CD45, CD34, CD19, HLA-DR, CD14, CD40, CD56, CD80, CD83, CD86, CD275	[36,202]
Chorionic plate MSCs	CD44, CD73, CD90, CD105, CD166, Oct-4, NANOG, Sox-2	CD14, CD19, CD34, CD45, HLA-DR	[203,204,205]
Decidua MSCs	CD44, CD90, CD105, CD146, CD166, HLA-ABC	CD40, CD80, CD83, CD86, HLA-DR	[193]
Umbilical cord MSCs	CD13, CD29, CD44, CD73, CD90, CD105, C10, CD49b-e, CD146, CD166, HLA-ABC, NANOG, Rex-1, Sox-2 Uncertain expression: Oct-3/4, SSEA-3, SSEA-4, STRO-1, TRA-1-60, TRA-1-81	CD14, CD31, CD34, CD45, CD51/61, CD64, CD106, HLA-DR	[206,207,208,209,210,211]
Amniotic fluid MSCs	CD73, CD90, CD105, Oct-4	CD45, CD34, CD31	[212]
Amniotic fluid stromal cells	CD29, CD44, CD73, CD90, CD105, SSEA-4, Oct-4, MHC-I, NANOG, SSEA-3, TRA-1-60, TRA-1-81	MHC-II, CD80, CD86	[167,213]
